# A phase 1–2, prospective, double blind, randomized study of the safety and efficacy of Sulfasalazine for the treatment of progressing malignant gliomas: study protocol of [ISRCTN45828668]

**DOI:** 10.1186/1471-2407-6-29

**Published:** 2006-01-31

**Authors:** Pierre A Robe, Didier Martin, Adelin Albert, Manuel Deprez, Alain Chariot, Vincent Bours

**Affiliations:** 1 Department of Neurosurgery, University of Liège, Domaine du Sart TIlman, B35, 4000 Liège, Belgium; 2 Department of Medical Statistics, University of Liège, Domaine du Sart TIlman, B35, 4000 Liège, Belgium; 3 Department of Pathology (Neuropathology), University of Liège, Domaine du Sart TIlman, B35, 4000 Liège, Belgium; 4 Department of Medical Chemistry University of Liège, Domaine du Sart TIlman, B35, 4000 Liège, Belgium; 5 Human Genetics. University of Liège, Domaine du Sart TIlman, B35, 4000 Liège, Belgium

## Abstract

**Background:**

The prognosis of patients suffering from WHO grade 3 and 4 astrocytic glioma remains poor despite surgery, radiation therapy and the use of current chemotherapy regimen. Indeed, the median survival of glioblastoma multiforme (WHO grade 4) patients is at best 14.6 month with only 26.5 percents of the patients still alive after 2 years and the median survival of anaplastic astrocytomas (WHO grade 3) is 19.2 month. Recent evidence suggests that the transcription factor NF-kappaB is constitutively expressed in malignant gliomas and that its inhibition by drugs like Sulfasalazine may block the growth of astrocytic tumors in vitro and in experimental models of malignant gliomas.

**Design:**

ULg_GBM_04/1 is a prospective, randomized, double blind single-center phase 1–2 study. A total of twenty patients with progressive malignant glioma despite surgery, radiation therapy and a first line of chemotherapy will be recruited and assigned to four dosage regimen of Sulfasalazine. This medication will be taken orally t.i.d. at a daily dose of 1.5–3–4 or 6 g, continuously until complete remission, evidence of progression or drug intolerance. Primary endpoints are drug safety in the setting of malignant gliomas and tumor response as measured according to MacDonald's criteria. An interim analysis of drug safety will be conducted after the inclusion of ten patients. The complete evaluation of primary endpoints will be conducted two years after the enrolment of the last patient or after the death of the last patient should this occur prematurely.

**Discussion:**

The aim of this study is to evaluate the safety and efficacy of Sulfasalazine as a treatment for recurring malignant gliomas. The safety and efficacy of this drug are analyzed as primary endpoints. Overall survival and progression-free survival are secondary endpoint.

## Background

The overall prognosis of malignant gliomas remains dismal despite recent improvements in their surgery, radiation therapy and chemotherapy [1-3]. Relapse usually occurs within 9 month after presentation, median survival barely reaches 1.6 years and less than 3% of the patients survive more than 3 years [4]. These survival data outline the urgent need to develop new treatment modalities and if possible to base these new treatments on the biochemical characteristics of tumor cells.

Recent evidence demonstrates that the NF-kappaB transcription factor is both over-expressed and constitutively activated in astrocytic malignant gliomas [5-8]. As in a variety of other tumor types, this activation contributes to the growth, survival and chemoresistance of tumor cells [9-11]. The potential benefit of NF-kappaB inhibition in the treatment of malignant tumors has been recently shown in multiple myeloma with the use proteasome inhibitor PS-341 [12,13].

Sulfalsalazine is an anti-inflammatory drug used for decades to treat inflammatory bowel disease and severe forms of rheumatoid arthritis [14]. It was shown to inhibit the activation of NF-kappaB in a variety of models [15,16]. We have also shown that Sulfasalazine induces apoptosis and inhibits the growth in glioblastoma cells but does not harm normal astrocytes. It also significantly reduces the *in vivo *growth of orthotopic xenografts of human malignant gliomas in nude mice [6].

Since there is a remarkable need for new, specific treatments for malignant gliomas and as the safety record of the drug Sulfasalazine is good, we have initiated a phase 1–2 clinical study of this drug foir the treatment of progressive or recurrent malignant astrocytic tumors. The working hypothesis is thus that Sulfaslazine should be well tolerated by patients with recurrent or progressive malignant glioma and could inhibit tumor progression.

## Design

### Study objectives

Primary objectives are twofold. First, this study will evaluate the maximal daily oral dose of Sulfaslazine that is tolerated by patients with recurrent or progressive malignant gliomas and will record the nature, frequency, possible causality and severity of adverse events that occur during treatment. The second primary objective is the assessment of any clinical and/ or radiological response of individual tumors to Sulfasalazine.

Overall and progression free survival following the initiation of Sulfasalazine treatment are secondary objectives of this study.

### Regulatory issues and monitoring

This study protocol was designed by the Departments of Neurosurgery and Human Genetics of the University Hospital of Liège, Belgium (PAR -principal investigator). The protocol was reviewed and approved by the Ethics Committee of the Faculty of Medicine of the University of Liège (IRB file number 2004/185). It also underwent review and approval by Belgian Federal Authorities (authorization reference 548/03/05) and was granted the European Trial database (EudraCT) number 2004-004392-11. It is sponsored by the Department of Neurosurgery, University Hospital of Liège. It is funded by the Leon Frederic Fund, which has neither access to the trial database nor right to review the results prior to their publicatrion, and is not otherwise involved in the study completion. The protocol has been listed on the International Standard Randomized Controlled Trial Number Register under number ISRCTN45828668.

An independent adverse events monitoring commission was set by the Ethics Committee and will review each serious adverse events and perform an interim analysis of the results for the first ten patients in order to evaluate the overall risk of the study and decide upon its further completion.

Study patients are covered by a medical liability insurance contracted by the University Hospital of Liege according to paragraph 29 of the Belgian Federal Law dated May 7, 2004. Medications are prepared and provided by the Liege University Hospital Pharmacy. They are prepared just before delivery to the patients and treatment refills are provided every 30 days during follow-up visits. The randomization table was set by the University of Liège Department of Medical Statistics using commercially available statistical software and directly provided to the hospital pharmacist. The pharmacist will provide the correlation between names and dosages to the independent review committee at the time of interim analysis and to the principal investigator at the time of final analysis.

### Overall design

ULg_GBM_04/1 is a phase 1–2, single center, prospective, randomized, double blind clinical study. To assess the maximal tolerated daily dose of Sulfasalazine, 4 groups of 5 patients will be given 1.5, 3, 4.5 or 6 grams of this drug. Sulfasalazine will be conditioned in similarly looking oral caps for each group, four of which will be taken orally three times per day. The randomization of patients between groups is provided by the department of Statistics of the Faculty of Medicine of the University of Liège and the drug dosage is communicated only to the hospital pharmacist who prepares and delivers the drug. In order to facilitate the interim analysis of the data by the review committee, the randomization algorithm was weighted so that 8 of the first 10 patients receive either the lowest or the highest drug dosage. Neither the investigators nor the patients are aware of the drug dosage that they provide/receive.

The medication is to be given continuously until complete remission or disease progression (as defined below) is observed, serious adverse events occur, the patients decides to withdraw from the study or regulatory authorities decide to halt the study.

### Inclusion criteria

Adult patients (age > 18 year) with recurrent or progressive WHO grade 3 or 4 astrocytic gliomas after surgery, standard radiation therapy and a first line of conventional chemotherapy (e.g., Temozolomide, CCNU or BCNU) are eligible for this study. Recurrence or progression prior to inclusion are based on MacDonald's criteria [17].

Patients are thoroughly informed about the nature of their disease, suspected prognosis, study background and objectives and potential alternative treatments. This information is provided both orally and in written form, prior to obtaining written informed consent from the patient. Upon consent, an experienced neuropathologist must confirm the diagnostic of grade 3 or 4 astrocytic glioma based on surgical specimen obtained previously (*confer supra*: standard treatment).

### Exclusion criteria

Patients will not enter the study if they present with anaplastic oligodendroglioma (WHO grade 3). Allergy to sulfa drugs, porphyria, G-6-PD deficiency and psychiatric disorder deemed incompatible with compliance to the study also contra-indicate enrollment in ULg_GBM_04/1. Creatinine > 15 mg/l, TGO> 200 UI/l or amylase > 150 UI/l are contra-indications as well.

ULg_GBM_04/1 is not open to pregnant or lactating women.

Finally, patients may not have received any other experimental medication within 30 days (and at least five drug half-lives) prior to inclusion in this study, and cannot concomitantly take mercaptopurine.

### Drug dosage and study duration

Sulfasalazine will be given orally three times a day at total doses of 1.5, 3, 4.5 or 6 grams. Four caps of drug are to be taken with each meal. Sulfaslazine is to be taken continuously until radiological evidence of tumor progression, complete remission, or the development of serious or intolerable adverse effects. The patient may at any moment decide to discontinue his participation to the study, although every effort will be made to be able to carry on the follow-up. Finally, the independent review committee may decide at any moment to end the study based on safety issues.

### Investigation schedule and evaluation of safety and efficacy

Patients with evidence of progression of WHO grade 3 or 4 astrocytic glioma will be considered for enrolment in EudraCT 2004-004392-11. Informed consent will be obtained and blood and urine will be obtained to assess renal, liver and pancreatic function as well as RBC, WBC and platelet counts. Beta-HCG will also be measured if the patient is a female. Pathology will be reviewed and if inclusion/ exclusion criteria are met, thorough medical history, clinical exam and an inclusion MRI will be performed. Sulfasalazine will be introduced within 10 days of this screening visit. Follow-up visits are scheduled every 15 days for 3 months to monitor adverse events and control the evolution of blood tests. Urinalysis, MRI and clinical follow-up are scheduled to occur every 30 days for the duration of Sulfasalazine treatment. Clinical follow-up is to be continued every 3 months after the end of Sulfasalazine treatment (Figure 1).

Safety will thus be evaluated by physical examination, blood tests and urinalysis on a regular basis. Adverse events will be recorded according to the NCI common toxicity criteria (CTCAE) version 3.0. Serious adverse events will be immediately reported to the independent review committee.

Efficacy will be assessed primarily as a measure of tumor volume changes. Tumor volume will be measured on gadolinium-enhanced T1 weighted images performed according to a standard protocol. Scans are scheduled to be made immediately prior to inclusion and every month after study enrollment. Volume changes will be evaluated according to Mac Donald's criteria [17]. Additional efficacy monitoring will be obtained through the follow-up of neurological deficits.

### Data analysis

Demographic, histological and clinical data of the patients will be described. Adverse effects will be reported qualitatively and classified according to body system, nature, severity and frequency. Unexpected causes of study drop-off will be reported and detailed. The incidence and severity of adverse effects will be correlated with drug dosage by means of contingency tests and linear regression.

MacDonald criteria-based tumor response will be reported and detailed with respect to its extent and duration.

Tumor volume changes will be described and analyzed in relation with drug dosage by means of contingency tests and if possible linear regression.

Patients overall survival (OS) and progression-free survival (PFS) will be described and stratified by dose of Sulfasalazine according to the Log-Rank method. Kaplan-Meier survival curves will also be provided.

## Competing interests

The author(s) declare that they have no competing interests.

## Authors' contributions

PAR, AC and VB are responsible for the study design, PAR and DM will recruit and treat the patients and manage the dataset, MD is responsible for the pathology review and AA is responsible for the statistical methodology and analysis.

## Pre-publication history

The pre-publication history for this paper can be accessed here:


